# Complete transfer of chirality in an intramolecular, thermal [2 + 2] cycloaddition of allene-ynes to form non-racemic spirooxindoles

**DOI:** 10.3762/bjoc.7.70

**Published:** 2011-05-12

**Authors:** Kay M Brummond, Joshua M Osbourn

**Affiliations:** 1 University of Pittsburgh, Department of Chemistry, Chevron Science Center, 219 Parkman Avenue, Pittsburgh, PA 15260, USA

**Keywords:** alkylidene cyclobutene, allene, allenyloxindole, chiral lanthanide shift reagent, chiral transfer

## Abstract

A thermal [2 + 2] cycloaddition reaction of allene-ynes has been used to transform chiral non-racemic allenyl oxindoles into chiral non-racemic spirooxindoles containing an alkylidene cyclobutene moiety. The enantiomeric excesses were determined by chiral lanthanide shift NMR analysis and the transfer of chiral information from the allene to the spirooxindole was found to be greater than 95%.

## Introduction

**Scheme 1:**
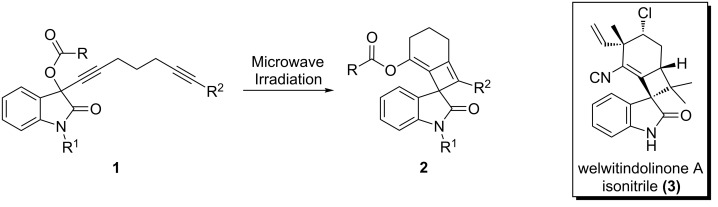
Conversion of propargyl acetate **1** to spirooxindole **2** containing the core framework of welwitindolinone A isonitrile (**3**).

The [2 + 2] cycloaddition reaction of allenes and alkynes provides rapid entry into synthetically challenging alkylidene cyclobutene ring systems. We, along with others, have demonstrated the intramolecular variant of this reaction under thermal conditions [1–2]. This thermally forbidden process is believed to proceed via a biradical intermediate mechanism, a conclusion supported by both computational and experimental studies [3]. Recently, the scope of this method has been expanded to the synthesis of spirooxindole-containing skeletons **2** in a two-step one-pot process from propargyl acetates **1** [4] (Scheme 1). Inspired by this rapid entry into the molecularly complex substructure **2**, and the structural similarity to welwitindolinone A isonitrile (**3**), we became interested in the synthesis of chiral non-racemic spirooxindoles for application to natural product synthesis [5–7]. Herein, we disclose preliminary results demonstrating a complete transfer of chiral information from a chiral non-racemic allene-yne to form an enantiomerically enriched spirooxindole in a [2 + 2] cycloaddition reaction.

## Findings

**Scheme 2:**
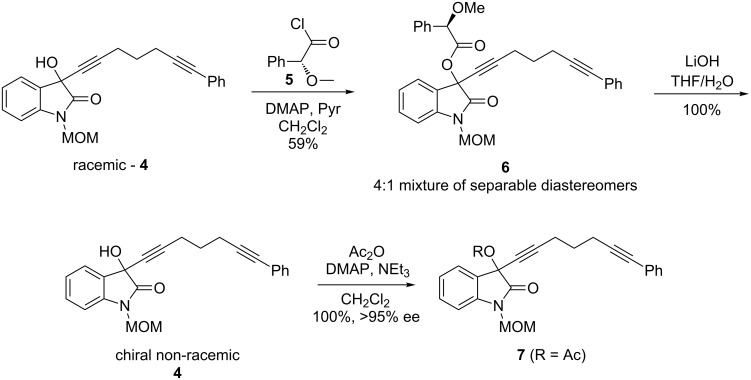
Preparation of enantiopure propargyl acetate **7** (R = Ac).

This study commenced with the preparation of the enantiopure propargyl acetate **7** [8]. Treatment of racemic propargyl alcohol **4** with the (*R*)*-*acid chloride **5**, DMAP, and pyridine resulted in propargyl ester **6** as a separable 4:1 mixture of diastereomers. Saponification of the major diastereomer of **6**, followed by acylation provided the propargyl acetate **7** in quantitative yield (Scheme 2).

**Figure 1:**
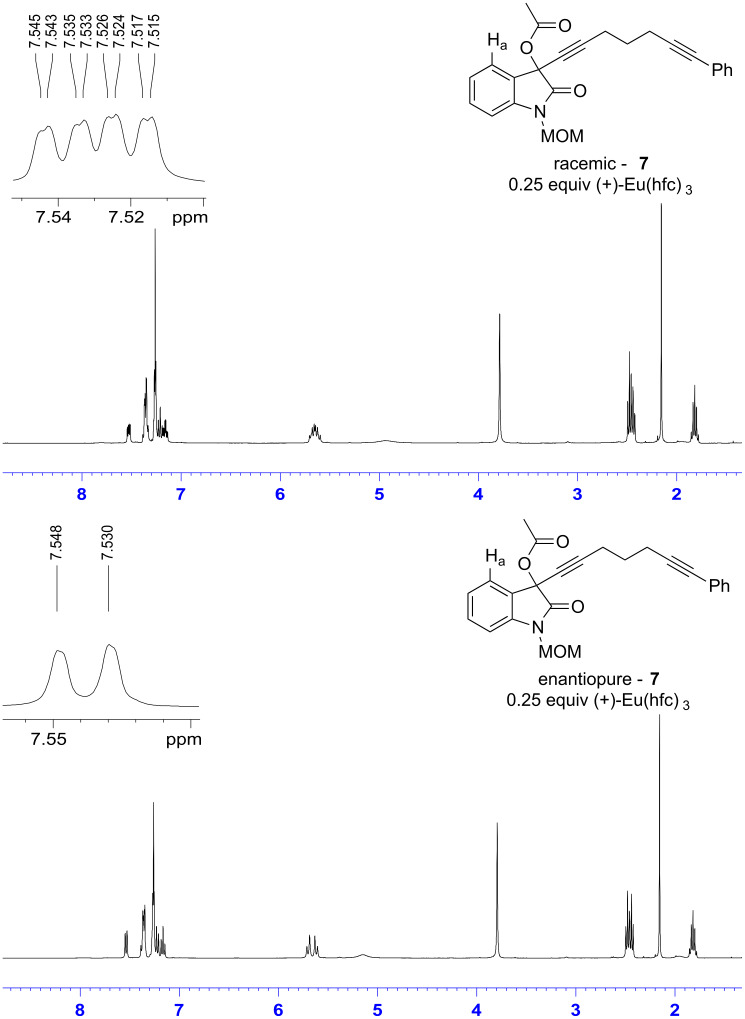
Chiral NMR shift analysis of propargyl acetate **7**.

The enantiomeric purity of propargyl acetate **7** was determined based on treatment of the compound with the chiral shift reagent (+)-Eu(hfc)_3_. Figure 1 shows the ^1^H NMR of the racemic as well as the enantiomerically enriched propargyl acetate upon treatment with the chiral shift reagent. Treating racemic acetate **7** with the chiral shift reagent enabled resolution of the resulting diastereomeric complexes, which was evidenced by the resonances for the aromatic proton labeled H_a_ in the spectrum shown below. The spectrum of racemic acetate **7** contains two doublets at δ 7.54 and 7.52, while the spectrum of enantiomerically enriched **7** shows only a single doublet at δ 7.54. Based on this result, the enantiomeric excess of enantiomerically enriched acetate **7** is greater than 95%.

Next, the focus turned to the conversion of enantiomerically enriched propargyl acetate **7** to an enantiomerically enriched allene **8**. It has been previously shown that delivery of an alkyl group from an organocuprate in an S_N_2’ fashion occurs with retention of chiral information in the resulting allene [9]; thus we began by screening cuprate conditions to form the desired allenyloxindole **8** (Table 1). In order to generate the allene, we examined various leaving groups (OMs, OMe, OAc), solvents (THF, Et_2_O), and cuprates (lower and higher order cyanocuprates). The reaction incorporating a mesylate as a leaving group was problematic due to substrate instability issues, even at low temperatures (Table 1, entry 1) and the substrates containing –OMe and –OAc leaving groups were unreactive toward the lower order cuprates (Table 1, entries 2–5). The optimal conditions were found using the propargylic acetate and the higher order cuprate, *t*-Bu_2_Cu(CN)Li_2_ at −78 °C which gave compound **8** (R’ = *tert*-butyl) in 49% yield (Table 1, entry 6). As a prelude to this proposed synthetic route, our attempts to thermally rearrange the chiral non-racemic propargyl acetate to form an enantiomerically enriched allenyl acetate followed by tandem [2 + 2] cycloaddition to provide directly a structure resembling compound **2**, gave the racemic spirooxindole product. This finding will be discussed in detail in a full account of this work.

**Table 1:** Screening conditions for the formation of allenyloxindole **8**.

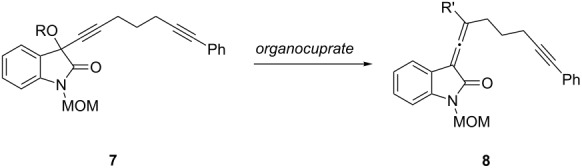						

Entry	R	Solvent	Temp	Cuprate	R’	Yield

1	Ms	THF	−45 °C	MeCu(CN)Li	Me	0%^a^
2	Me	Et_2_O	0 to 35 °C	MeCu(CN)Li	Me	0%^b^
3	Ac	Et_2_O	0 °C	MeCu(CN)Li	Me	0%^b^
4	Ac	THF	−78 °C to rt	MeCu(CN)Li	Me	0%^c^
5	Ac	Et_2_O	−78 °C	*t*-BuCu(CN)Li	*t*-Bu	0%^b^
6	Ac	THF	−78 °C	*t*-Bu_2_Cu(CN)Li_2_	*t*-Bu	49%^d^

^a^Complete decomposition of the mesylate (generated in situ) was observed prior to cuprate addition. ^b^Complete recovery of starting material. ^c^Starting material was recovered in addition to the deacylation product. ^d^The product of a second addition of the tert-butyl group to the central carbon of the allene was also isolated in 21% yield.

**Figure 2:**
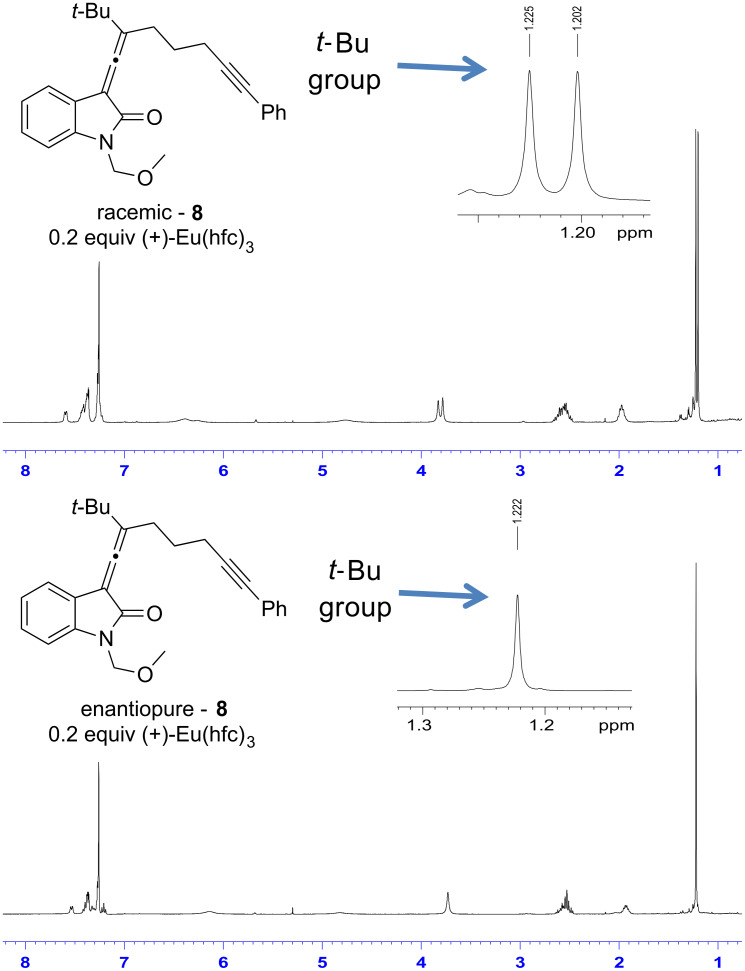
Chiral NMR shift analysis of allenyloxindole **8**.

To examine the chiral transfer from the propargylic acetate **7** to the allenyloxindole **8**, a chiral ^1^H NMR shift analysis was performed using (+)-Eu(hfc)_3_. The NMR spectra of the racemic compound **8** as well as the enantiomerically enriched compound **8** are shown in Figure 2. In the case of the racemic compound, the resonances for the *tert*-butyl groups of the diastereomeric complexes are split into two distinct signals in the presence of the shift reagent; one singlet at δ 1.23 ppm and a second singlet at δ 1.20 ppm. The enantiomerically enriched compound **8** shows one singlet, corresponding to the resonance for the *tert*-butyl group at δ 1.22 ppm; thus the enantiomeric excess of allenyloxindole **8** is greater than 95%.

**Scheme 3:**
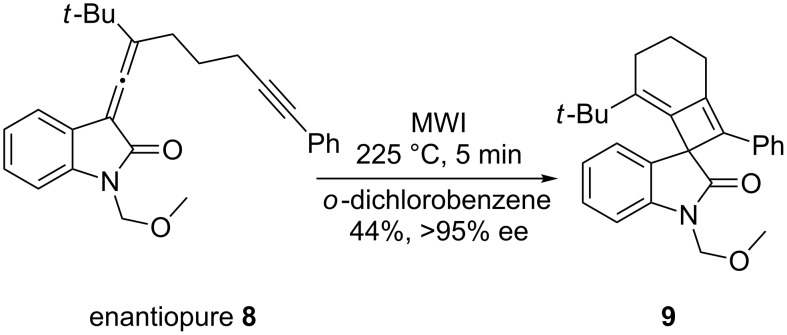
Microwave irradiation of allenyloxindole **8**.

With the enantiomerically enriched allene-yne **8** in hand, we were poised to test the transfer of chiral information under thermal [2 + 2] reaction conditions. Irradiation of **8** in *o*-dichlorobenzene with microwaves for 5 min at 225 °C provided the desired spirooxindole **9** in 44% yield (Scheme 3). Despite the somewhat low yield, the reaction is clean as judged by TLC with the exception of some baseline material. Spirooxindole **9** was purified by column chromatography and the transfer of chiral information was determined using chiral ^1^H NMR shift analysis.

**Figure 3:**
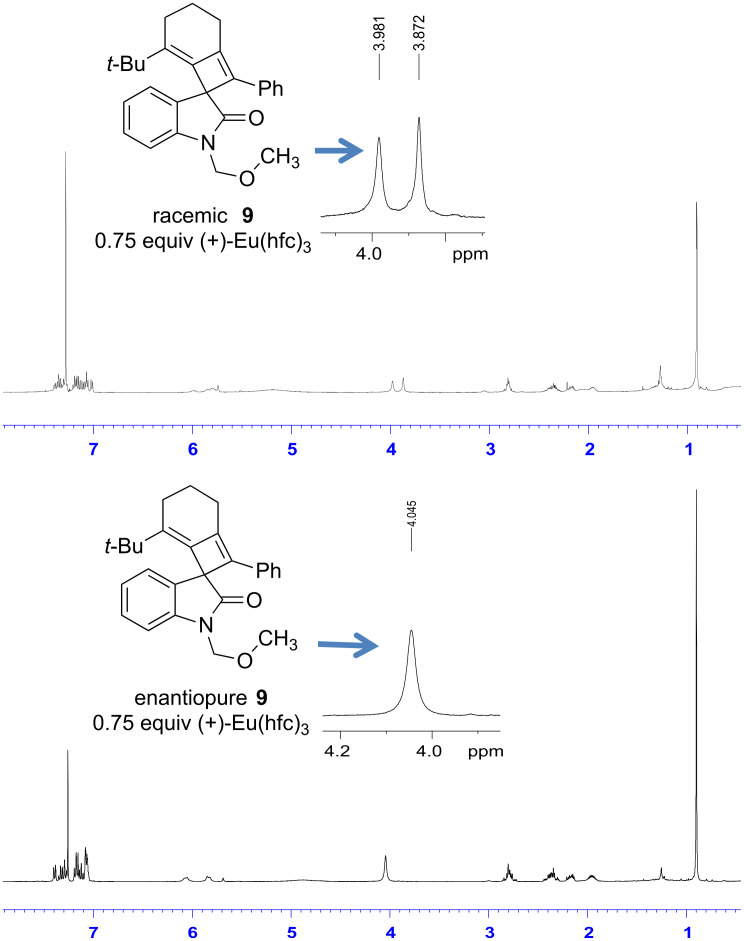
Chiral NMR shift analysis of spirooxindole **9**.

Spirooxindole **9** was treated with 0.75 equiv of (+)-Eu(hfc)_3_ in CDCl_3_. The resulting diastereomeric complexes were resolved based upon the resonances at δ 3.98 and δ 3.87 ppm, which are peaks that correspond to the methyl in the MOM group on the oxindole nitrogen. For the enantiomerically enriched compound **9**, only a single resonance is observed at δ 4.05 ppm in the presence of the chiral shift reagent. Based on this analysis, the product was formed with greater than 95% enantiomeric excess (Figure 3).

**Figure 4:**
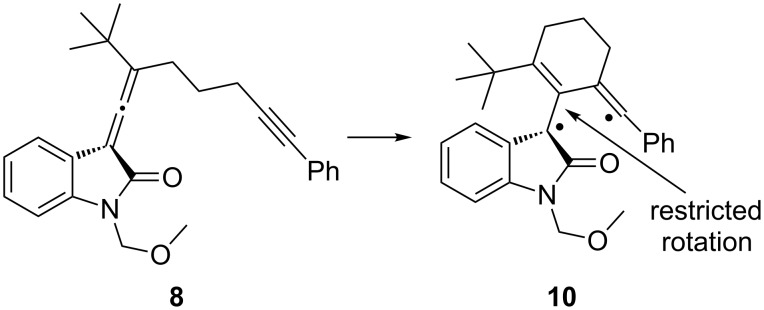
Thermally generated biradical intermediate **10**.

Our working hypothesis for the mechanism for transfer of chiral information from the allene to the spirooxindole-containing cyclobutene is that the reaction still proceeds through the thermally generated biradical intermediate, but the *tert*-butyl group hinders rotation around the carbon–carbon bond as shown in Figure 4, thus slowing racemization of the resulting radical containing carbon. This hypothesis is supported by a report by Pasto, where transfer of chiral information was incomplete in a thermal, intermolecular [2 + 2] cycloaddition reaction between 2,3-pentadiene and methyl propiolate [10].

In summary, there are only a few general methods to prepare carbocyclic spirooxindoles non-racemically [11–14]; we have demonstrated the first thermal, intramolecular [2 + 2] cycloaddition reaction of an allene-yne that generates a chiral non-racemic spirooxindole from a chiral non-racemic allene. Furthermore, this reaction could also be applicable in the enantioselective synthesis of natural products that contain a spirooxindole core, such as welwitindolinone A isonitrile. We are currently working to expand the scope of this chirality transfer to other allenyl systems possessing less bulky and/or traceless groups.

## Supporting Information

File 1 General methods, experimental and spectral data for all new compounds.
