# Supplementary material for: Complete transfer of chirality in an intramolecular, thermal [2 + 2] cycloaddition of allene-ynes to form non-racemic spirooxindoles
Source: Beilstein J Org Chem. 2011 May 12;7:601–5. doi: 10.3762/bjoc.7.70 (PMC3107521; doi:10.3762/bjoc.7.70)

Supporting Information

for

Complete transfer of chirality in an intramolecular, thermal [2 + 2] cycloaddition of allene-ynes to form non-racemic spirooxindoles

Kay M. Brummond* and Joshua M. Osbourn

Address: University of Pittsburgh, Department of Chemistry, Chevron Science Center, 219 Parkman Avenue, Pittsburgh, PA 15260, USA

Email: Kay M. Brummond - kbrummon@pitt.edu

* Corresponding author

General methods, experimental and spectral data for all new compounds.

General Methods

Unless otherwise noted, all reactions were performed in flame-dried glassware under an inert atmosphere of dry nitrogen. All commercially available compounds were purchased from Aldrich Chemical Co., GFS Chemicals, Strem Chemicals, Acros Organics and Advanced Chemtech, and used as received. The reaction solvents tetrahydrofuran (THF), diethyl ether (Et_2O), and dichloromethane (CH_2Cl_2) were purified by passing through alumina using the Sol-Tek ST-002 solvent purification system. Triethylamine (NEt_3) and 1,2-dichlorobenzene were freshly distilled from CaH_2 prior to use. Purification of the compounds by flash column chromatography was performed on silica gel (32-63 µm particle size, 60 Å pore size) purchased from Sorbent Technologies. TLC analyses were performed on EMD Chemicals Silica Gel 60 F_{254} glass plates (250 µm thickness). ^{1}H NMR and ^{13}C NMR spectra were recorded on Bruker Avance 300 MHz, 400 MHz, or 500 MHz spectrometers. Spectra were referenced to residual chloroform (7.26 ppm, ^{1}H, 77.0 ppm, ^{13}C). Chemical shifts are reported in ppm, multiplicities are indicated by s (singlet), d (doublet), t (triplet), q (quartet), quin (quintet), sextet (sextet), and m (multiplet). Coupling constants, *J*, are reported in Hertz. All NMR spectra were obtained at room temperature. IR spectra were obtained using a Nicolet Avatar E.S.P. 360 FT-IR. Optical rotations were determined on a Perkin-Elmer polarimeter in a 0.5 mm cell with a 1 cm path length. EI mass spectroscopy was performed on a Micromass Autospec high-resolution mass spectrometer. All microwave-mediated reactions were carried out with a Biotage Initiator™ Exp microwave synthesizer. The microwave parameters were set to variable power, constant temperature, with the fixed hold time set to on. The microwave reactions were carried out in 0.5–2 mL or 2–5 mL Biotage™ microwave vials. The chiral lanthanide shift reagent (+)-Eu(hfc)$_3$ was purchased from Aldrich and was used as received.

General Method for the Chiral Shift Analysis

A known amount of racemic substrate was dissolved in 0.5 mL $CDCl_3$ + 0.05% TMS. To this solution, the chiral lanthanide shift reagent (+)-Eu(hfc)$_3$ (0.05 equiv) in 0.05 mL $CDCl_3$ was added. A ^{1}H NMR spectrum was then obtained. The shift reagent was continually added in 0.05 equiv. portions until complete resolution of the two diastereomeric complexes was observed in the ^{1}H NMR spectrum. Once appropriate conditions were determined on the racemic substrate, the chiral shift analysis was performed on the enantiomerically enriched substrate with exactly the same quantity of substrate, shift reagent, and solvent.

Literature Preparations

Preparation of propargyl alcohol **4** was reported previously by our group [1]. Acid chloride **5** was prepared from the corresponding carboxylic acid via the method of Hoye [2].

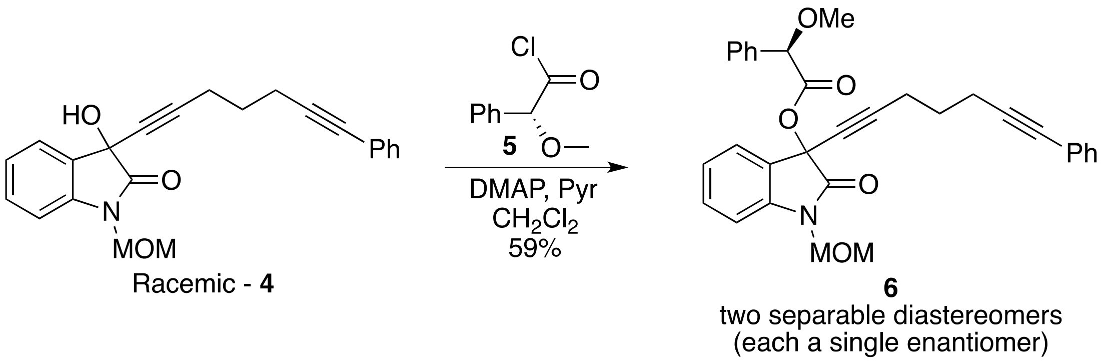

(2*R*)-1-(methoxymethyl)-2-oxo-3-(7-phenylhepta-1,6-diyn-1-yl)indolin-3-yl 2-methoxy-2-phenylacetate (6). A flame-dried single necked 25 mL round bottomed flask was charged with the acid chloride **5** (421 mg, 2.28 mmol) and dichloromethane (7.3 mL). Propargyl alcohol **4** (654 mg, 1.82 mmol) was added followed by pyridine (0.293 mL, 3.64 mmol), and finally 4-dimethylaminopyridine (22.2 mg, 0.182 mmol). The reaction was maintained at room temperature for 2 h by which time all the starting material had been consumed based on TLC. The reaction was quenched by adding saturated sodium bicarbonate solution (5 mL). The layers were separated and the aqueous layer was extracted with dichloromethane (3 x 5 mL). The combined organic layers were washed with brine (5 mL), dried over magnesium sulfate and concentrated. The crude material was purified by silica gel flash chromatography eluting with a gradient of 100% toluene – 5% ethyl acetate/toluene to afford 244 mg of the less polar diastereomer, 95 mg of the more polar diastereomer, and 205 mg of a mixture in a combined yield of 59%.

Data for **6**: (JMO6-158)

Less Polar Diastereomer:

^{1}H NMR (400 MHz, $CDCl_3$)

7.41 – 7.24 (m, 12H), 7.06 – 7.02 (m, 2H), 5.15 (d, *J* = 11.2 Hz, 1H), 5.11 (d, *J* = 11.2 Hz, 1H), 4.81 (s, 1H), 3.40 (s, 3H), 3.38 (s, 3H), 2.43 (t, *J* = 7.2 Hz, 2H), 2.39 (t, *J* = 7.2 Hz, 2H), 1.77 (quin, *J* = 6.8 Hz, 2H) ppm

^{13}C NMR (100 MHz, $CDCl_3$)

170.6, 168.6, 141.6, 135.1, 131.5 (2C), 130.5, 128.9, 128.6 (2C), 128.2 (2C), 127.6, 127.2 (2C), 126.3, 123.7, 123.6, 123.4, 110.2, 89.3, 88.7, 82.3, 81.3, 74.0, 73.9, 72.0, 57.5, 56.5, 27.1, 18.5, 18.2 ppm

IR 2937, 2360, 2244, 1746, 1612 cm^{-1}

MS ESI+ *m/z* (%)

1038 ($[2M+Na+H]^+$, 75), 1037 ($[2M+Na]^+$, 100), 530 ($[M+Na]^+$, 75), 380 (55), 298 (55), 280 (60), 267 (95)

HRMS ESI+: $[M+Na]^+$ $C_{32}H_{29}NO_5Na$

Calculated 530.1943; Found 530.1981

TLC R_f = 0.55 (4 elutions of 20% EtOAc/hexanes) [silica gel, UV]

More Polar Diastereomer

^{1}H NMR (400 MHz, $CDCl_3$)

7.37 – 7.33 (m, 8H), 7.29 – 7.25 (m, 3H), 7.02 (d, *J* = 8 Hz, 1H), 6.88 (dt, *J* = 0.8, 7.6 Hz, 1H), 6.69 (dd, *J* = 0.8, 7.6 Hz, 1H), 5.18 (d, *J* = 11.2 Hz, 1H), 5.14 (d, *J* = 10.8 Hz, 1H), 4.89 (s, 1H), 3.42 (s, 3H), 3.38 (s, 3H), 2.43 (t, *J* = 7.2 Hz, 2H), 2.39 (t, *J* = 7.2 Hz, 2H), 1.77 (quin, *J* = 6.8 Hz, 2H) ppm

^{13}C NMR (100 MHz, $CDCl_3$)

171.0, 168.2, 141.7, 135.2, 131.5 (2C), 130.5, 128.9, 128.8, 128.6 (2C), 128.2 (2C), 127.7, 127.5 (2C), 125.6, 123.5, 122.9, 110.2, 89.2, 88.6, 81.6, 81.3, 73.9 (2C), 72.1, 57.5, 56.6, 27.1, 18.5, 18.1 ppm

IR 2937, 2244, 1746, 1613 cm^{-1}

MS ESI+ *m/z* (%)

530 (100), 527 (63), 365 (50), 280 (33), 267 (42)

HRMS ESI+: $[M+Na]^+$ $C_{32}H_{29}NO_5Na$

Calculated 530.1943; Found 530.1896

TLC R_f = 0.50 (4 elutions of 20% EtOAc/hexanes) [silica gel, UV]

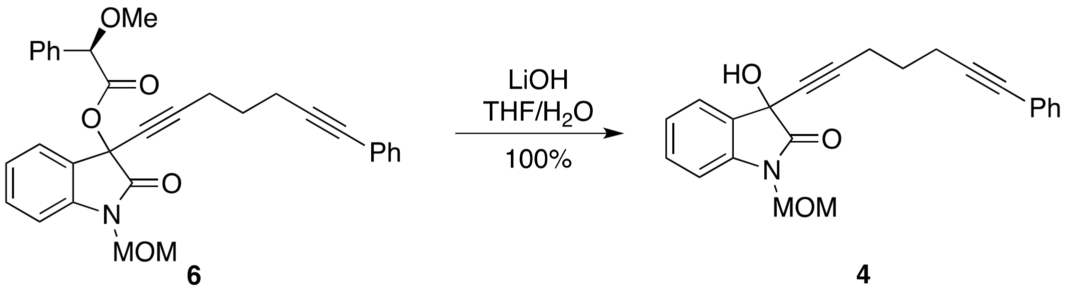

6
(less polar diastereomer)

4

3-hydroxy-1-(methoxymethyl)-3-(7-phenylhepta-1,6-diyn-1-yl)indolin-2-one (4). A 10 mL round bottomed flask was charged with ester **6** (less polar diastereomer) (140 mg, 0.276 mmol), tetrahydrofuran (1.8 mL), and water (1.8 mL). Lithium hydroxide monohydrate (23.2 mg, 0.552 mmol) was added in one portion and the reaction was left to stir at room temperature for 12 h at which time additional lithium hydroxide monohydrate (23.2 mg, 0.552 mmol) was added and the reaction mixture was maintained at room temperature for a further 2 h. The reaction mixture was then transferred to a separatory funnel. The reaction flask was rinsed with diethyl ether (5 mL) and water (5 mL) and the washings also transferred to the separatory funnel. The layers were separated and the aqueous layer was extracted with diethyl ether (3 x 3 mL). The combined organic layers were dried over anhydrous magnesium sulfate and concentrated to provide 98.0 mg of propargyl alcohol **4** in 100% yield as a cream colored solid. The product was used without further purification.

Data for **4**: (JMO6-160)

^{1}H NMR (300 MHz, $CDCl_3$)
7.57 (dd, J = 0.9, 7.5 Hz, 1H), 7.40 – 7.35 (m, 3H), 7.31 – 7.27 (m, 3H), 7.19 (dt, J = 0.6, 7.5 Hz, 1H), 7.06 (d, J = 7.5 Hz, 1H), 5.16 (d, J = 11.1 Hz, 1H), 5.12 (d, J = 11.1 Hz, 1H), 3.53 (s, 1H), 3.37 (s, 3H), 2.50 (t, J = 6.9 Hz, 2H), 2.44 (t, J = 6.9 Hz, 2H), 1.82 (quin, J = 7.2 Hz, 2H) ppm

^{13}C NMR (75 MHz, $CDCl_3$)
196.7, 174.7, 141.2, 131.5 (2C), 130.4, 128.7, 128.2 (2C), 127.7, 124.6, 124.1, 123.6, 110.3, 88.7, 87.3, 81.3, 71.7, 69.4, 56.3, 27.3, 18.5, 18.1 ppm

TLC R_f = 0.11 (20% EtOAc/hexanes) [silica gel, UV]

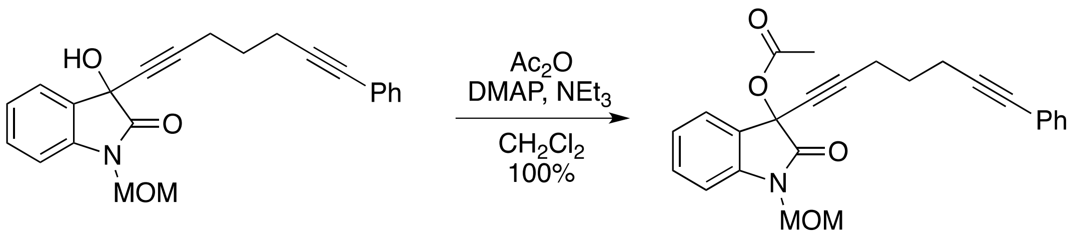

4
(Chiral Non-Racemic)

7
(>95% ee)

1-(methoxymethyl)-2-oxo-3-(7-phenylhepta-1,6-diyn-1-yl)indolin-3-yl acetate (7). A 2-dram vial was charged with propargyl alcohol **4** (98 mg, 0.273 mmol) and dichloromethane (1.0 mL). 4-Dimethylaminipyridine (3.3 mg, 0.0273 mmol), triethylamine (137 µL, 0.983 mmol), and finally acetic anhydride (62 µL, 0.655 mmol) were added. The reaction was stirred at room temperature for 2 h by which time all starting material had been consumed based on TLC. The reaction was quenched by adding saturated aqueous sodium bicarbonate (1.0 mL). The layers were separated and the aqueous layer was extracted with dichloromethane (2 x 1 mL). The combined organic layers were dried over anhydrous magnesium sulfate and concentrated. The crude material was purified by silica gel flash chromatography with 20% ethyl acetate/hexanes as elutent. The fractions containing the desired product were concentrated to provide 110 mg of propargyl acetate **7** as a lightly colored oil in 100% yield. Propargyl acetate **7** was determined to be >95% ee based on ^{1}H NMR in the presence of a chiral lanthanide shift reagent. NMR Sample: 42 mg **7**, 15.5 mg (+)-Eu(hfc)$_3$, 0.5 mL $CDCl_3$ + 0.05% TMS. *See text for spectra of the racemic and enantioenriched compounds in the presence of the chiral shift reagent.*

Data for **7**: (JMO6-162)

^{1}H NMR (300 MHz, $CDCl_3$)
7.44 (d, *J* = 7.5 Hz, 1H), 7.39 – 7.34 (m, 3H), 7.29 – 7.27 (m, 3H), 7.14 (t, *J* = 7.5 Hz, 1H), 7.08 (d, *J* = 8.1 Hz, 1H), 5.22 (d, *J* = 11.1 Hz, 1H), 5.15 (d, *J* = 11.1 Hz, 1H), 3.43 (s, 3H), 2.48 (t, *J* = 7.2 Hz, 2H), 2.44 (t, *J* = 7.2 Hz, 2H), 2.11 (s, 3H), 1.82 (quin, *J* = 6.9 Hz, 2H) ppm

^{13}C NMR (75 MHz, $CDCl_3$)
171.3, 168.5, 141.6, 131.4 (2C), 130.4, 128.1 (2C), 127.6, 126.6, 123.7, 123.5, 123.2, 110.2, 88.8, 88.6, 81.3, 74.2, 73.4, 72.0, 56.5, 27.1, 20.6, 18.5, 18.1 ppm

TLC R_f = 0.24 (20% EtOAc/hexanes) [silica gel, UV, *p*-anisaldehyde stain]

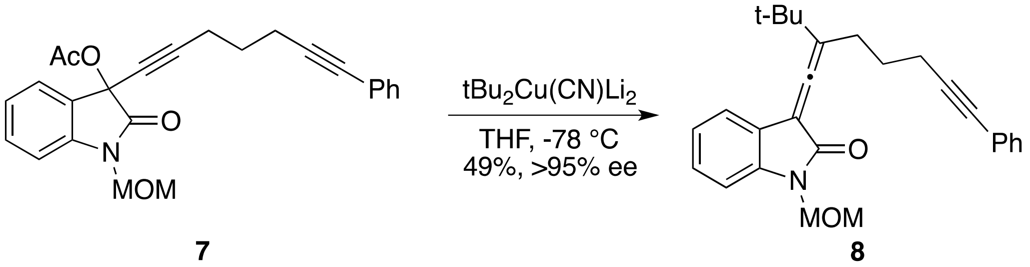

3-(2-*tert*-butyl-7-phenylhept-1-en-6-yn-1-ylidene)-1-(methoxymethyl)indolin-2-one (8). A 25 mL round bottomed flask was charged with copper(I)cyanide (83.8 mg, 0.935 mmol). The flask was equipped with a magnetic stirring bar and a septum and was gently flame dried under vacuum. Upon heating, the copper(I)cyanide changed from a light green color to a light tan color. *Caution: Care must be taken when working with copper(I)cyanide; one must avoid any conditions that could generate hydrogen cyanide.* The flask was cooled under a positive atmosphere of nitrogen. Tetrahydrofuran (6.7 mL) was then added and the heterogeneous mixture was cooled to −78 °C. *Tert*-butyllithium (1.1 mL of a 1.7 M pentane solution, 1.87 mmol) was then added dropwise over a 20 min period. After stirring at −78 °C for 30 min, the solution became pale yellow and homogeneous. Propargyl acetate **7** (150 mg, 0.374 mmol) in tetrahydrofuran (3.7 mL) was added to the solution. After 10 min at −78 °C, all the starting material had been consumed based on TLC. To quench the reaction, 5 mL of a 9:1 saturated aqueous ammonium chloride: ammonium hydroxide solution was added. The solution was stirred for 1h during which time it became deep blue in color. The aqueous layer was extracted with diethyl ether (3 x 2 mL). The combined organic layers were dried over magnesium sulfate and concentrated. The crude material was purified by silica gel flash chromatography eluting with a gradient of 10–19% ethyl acetate/hexanes. The fractions containing the desired product were concentrated to provide allene **8** (72.8 mg, 49%) as a light yellow oil. A small amount of product resulting from a second addition of a *tert*-butyl group to the central carbon of the allene (R_f = 0.69, 20% ethyl acetate/hexanes) was also isolated. *See text for spectra of the racemic and enantioenriched compounds in the presence of the chiral shift reagent.*

Data for **8**: (JMO7-100)

^{1}H NMR (400 MHz, $CDCl_3$)

7.37 – 7.34 (m, 2H), 7.30 – 7.23 (m, 5H), 7.07 (dt, J = 7.6, 0.8 Hz, 1H), 7.03 (d, J = 8.0 Hz, 1H), 5.18 (s, 2H), 3.36 (s, 3H), 2.48 (m, 4H), 1.85 – 1.70 (m, 2H), 1.22 (s, 9H) ppm

^{13}C NMR (100 MHz, $CDCl_3$)

203.4, 168.4, 140.0, 131.5 (2C), 128.4, 128.1 (2C), 127.5, 124.5, 123.8, 122.7, 122.3, 121.3, 109.4, 102.2, 89.4, 81.2, 71.4, 56.3, 36.0, 29.4 (3C), 26.8, 26.7, 18.8 ppm

IR (thin film)

2962, 1945, 1711, 1610 cm^{-1}

HRMS TOF MA ESI+: $C_{27}H_{29}NO_2Na$

Calculated: 422.2096 Found: 422.2122

Rotation $[\alpha]_D^{25}$ = −3.64 (c 1.1, $CHCl_3$)

TLC R_f = 0.53 (20% EtOAc/hexanes) [silica gel, UV, *p*-anisaldehyde stain]

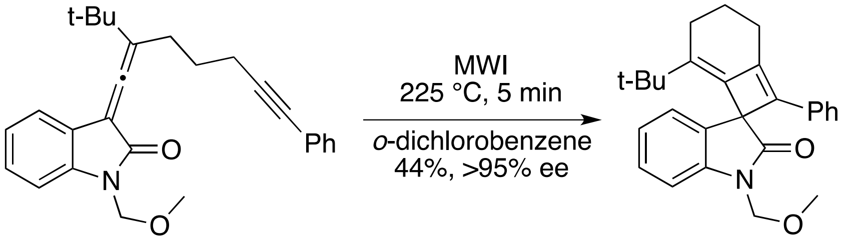

Chiral, Non-Racemic **8** **9**

5-*tert*-butyl-1'-(methoxymethyl)-8-phenylspiro[bicyclo[4.2.0]octa[1(8),5]diene-7,3'-indolin]-2'-one (9). A 0.5–2 mL Biotage microwave vial containing a magnetic stirring bar was charged with allene **8** (10 mg, 0.025 mmol) and *o*-dichlorobenzene (0.6 mL). The vial was capped and irradiated with microwaves for 5 min at 225 °C. The reaction mixture was loaded onto a silica gel flash column and eluted with hexanes followed by a gradient of 5–17% ethyl acetate/hexanes. The fractions containing the desired product were concentrated to provide 4.4 mg of spirooxindole **9** in 44% yield and >95% ee as a lightly colored oil.

Data for **9**: (JMO7-124)

^{1}H NMR (400 MHz, $CDCl_3$)

7.31 (dt, J = 7.7, 1.2 Hz, 1H), 7.21 (dd, J = 7.4, 0.7 Hz, 1H), 7.15-7.11 (m, 3H), 7.07-7.02 (m, 2H), 6.87-6.85 (m, 2H), 5.27 (d, J = 10.9 Hz, 1H), 5.18

(d, J = 10.9 Hz, 1H), 3.39 (s, 3H), 2.72-2.68 (m, 2H), 2.32-2.19 (m, 2H), 2.12-2.03 (m, 1H), 1.91-1.81 (m, 1H), 0.78 (s, 9H) ppm

^{13}C NMR — (100 MHz, $CDCl_3$)
177.3, 149.8, 141.8, 137.6, 133.3, 132.6, 132.0, 129.2, 128.5 (3C), 126.7, 125.2 (2C), 123.6, 123.2, 109.5, 77.2, 71.8, 56.9, 34.8, 28.0 (3C), 25.8, 24.3, 23.1 ppm

Rotation — $[\alpha]_D^{25}$ = −3.64 (c 1.1, $CHCl_3$)

TLC — R_f = 0.51 (2 elutions of 5% EtOAc/hexanes) [silica gel, UV, *p*-anisaldehyde stain]

References

1. Brummond, K. M., Osbourn, J. M. *Beilstein J. Org. Chem.* **2010**, *6*, No 33.
2. Hoye, T. R., Erickson, S. E., Erickson-Birkedahl, S. L., Hale, C. R. H., Izgu, E. C., Mayer, M. J., Notz, P. K., Renner, M. K. *Org. Lett.* **2010**, *12*, 1768.

JMO6-158 - Diastereomer 1
CDCl3 + TMS
NMR 400B

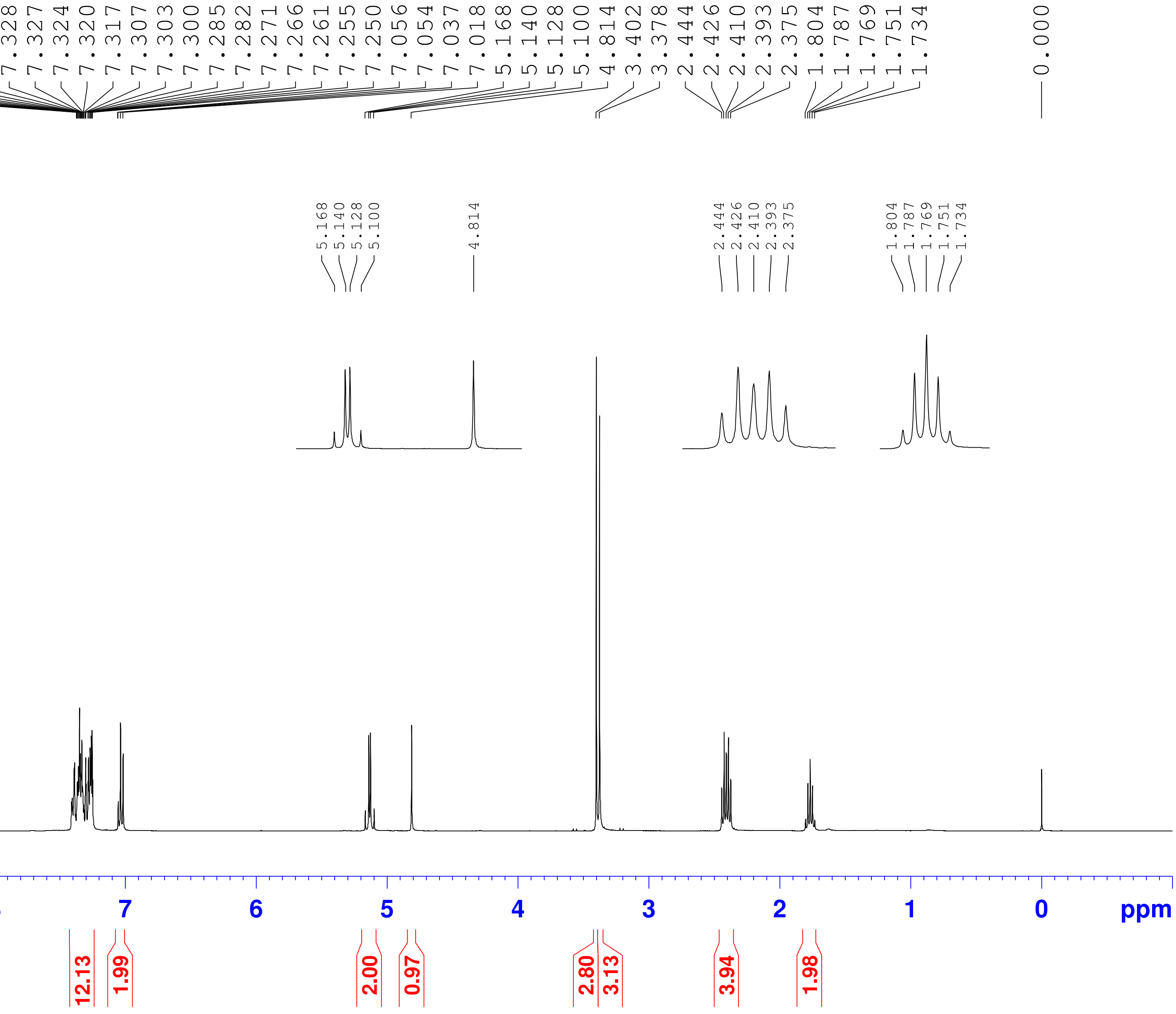

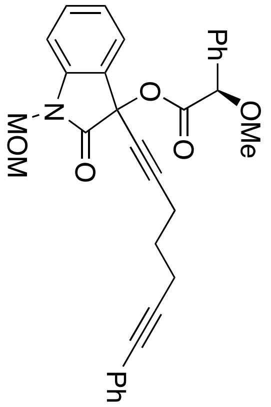

6

JMO6-158 - Diastereomer 1
CDCl3 + TMS
NMR 400B

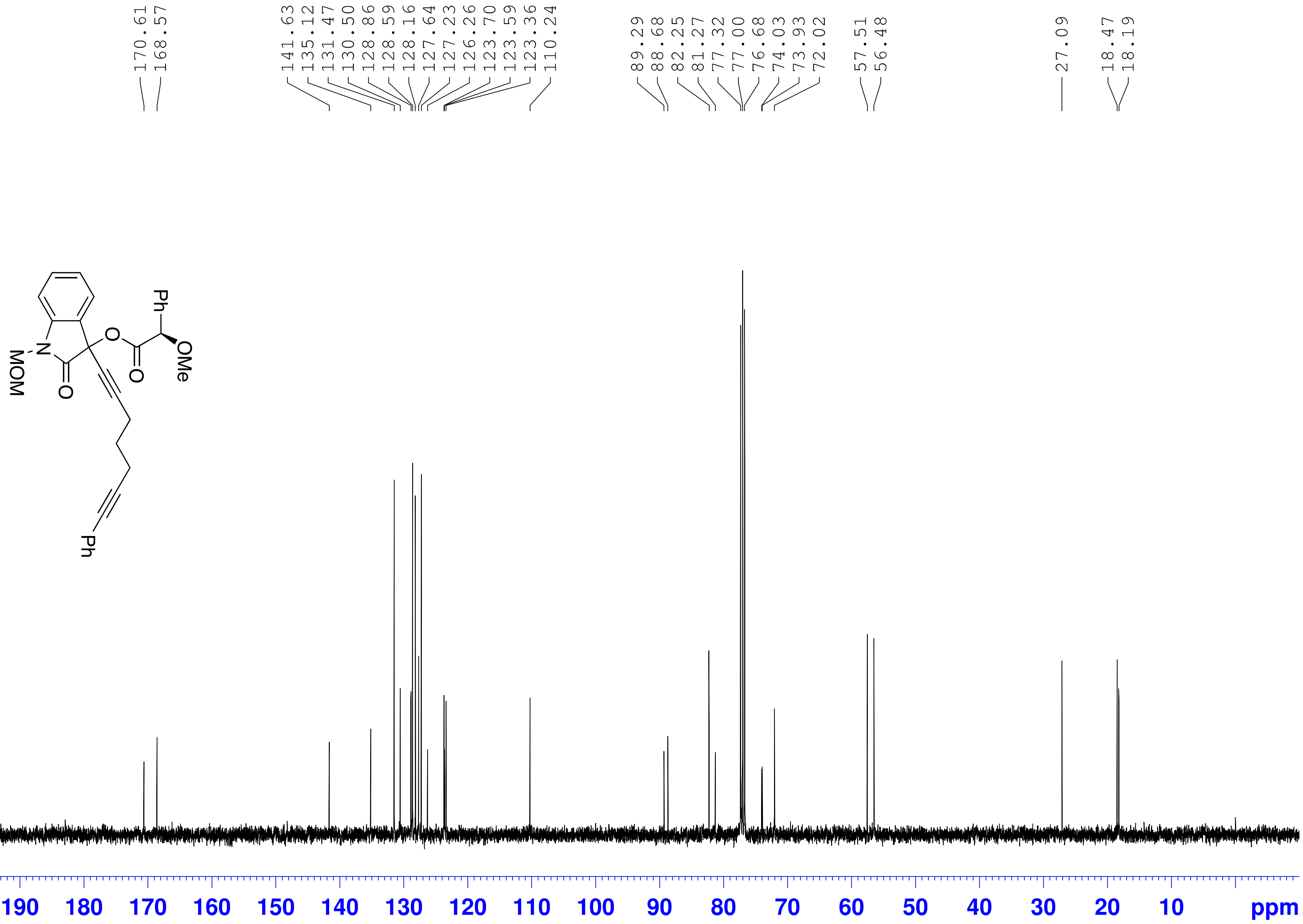

6

JMO6-158 - Diastereomer 2
CDCl3 + TMS
NMR 400B

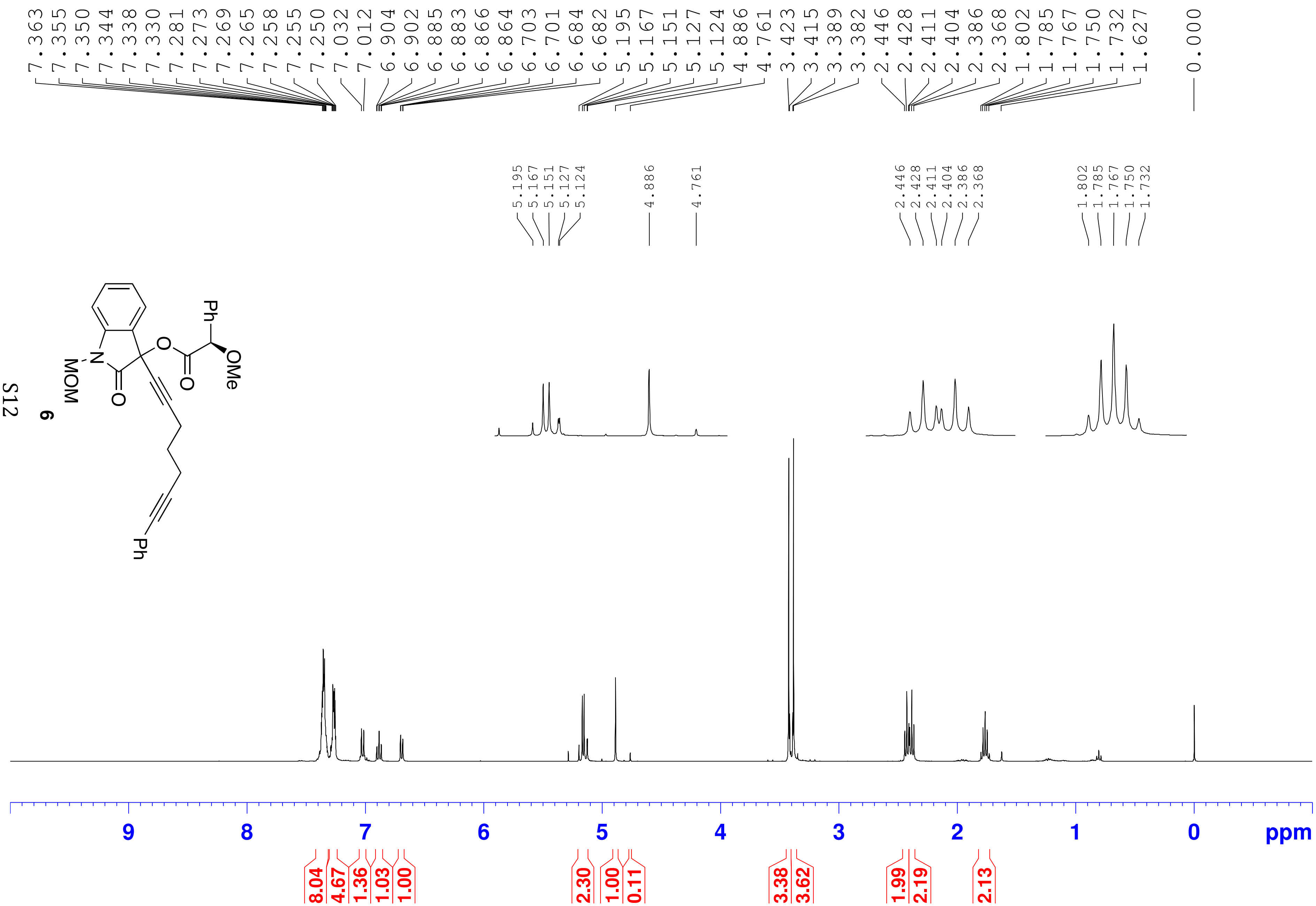

6

JMO6-158 - Diastereomer 2
CDCl3 + TMS
NMR 400B

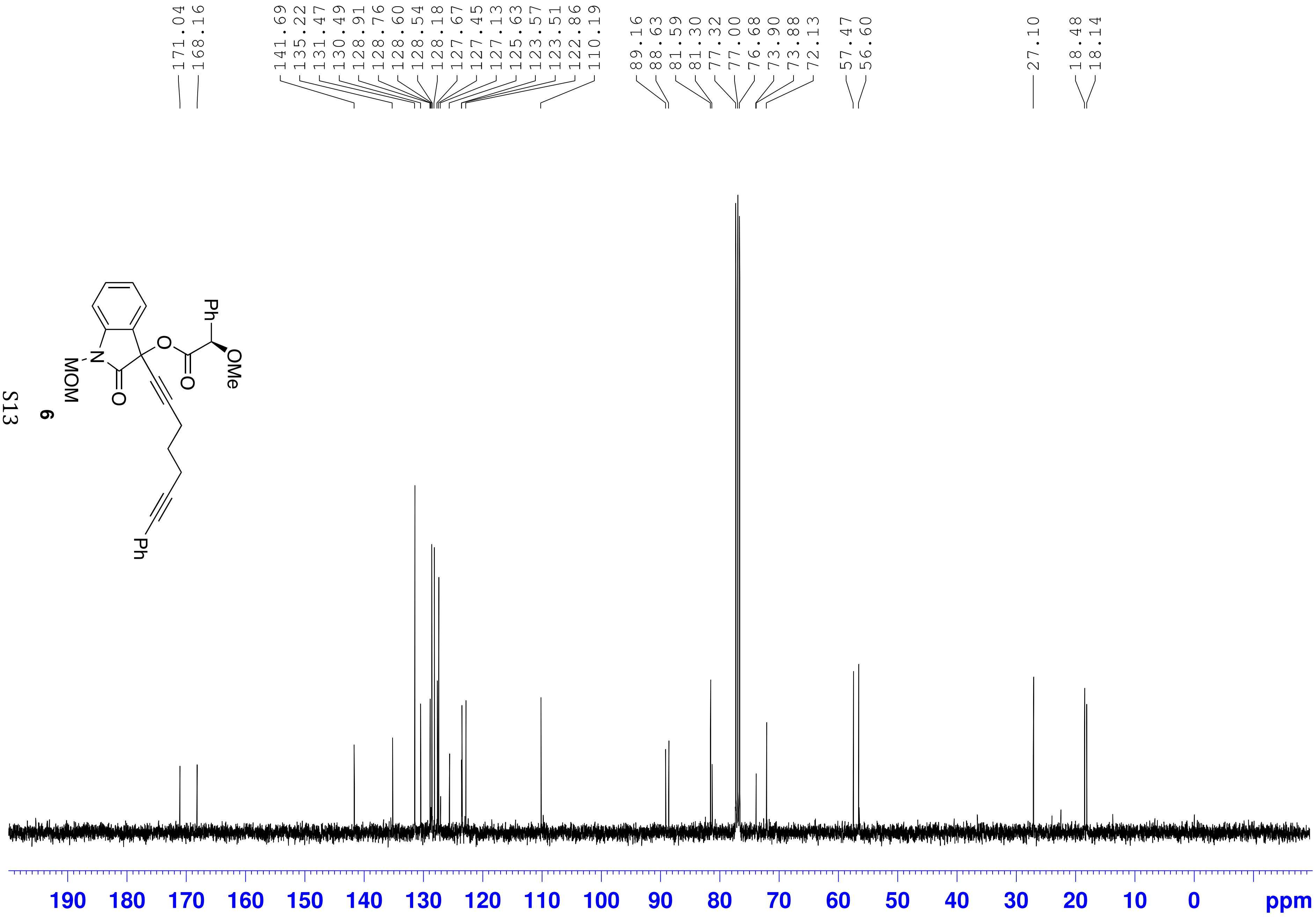

6

JMO6-160 Crude
NMR 300

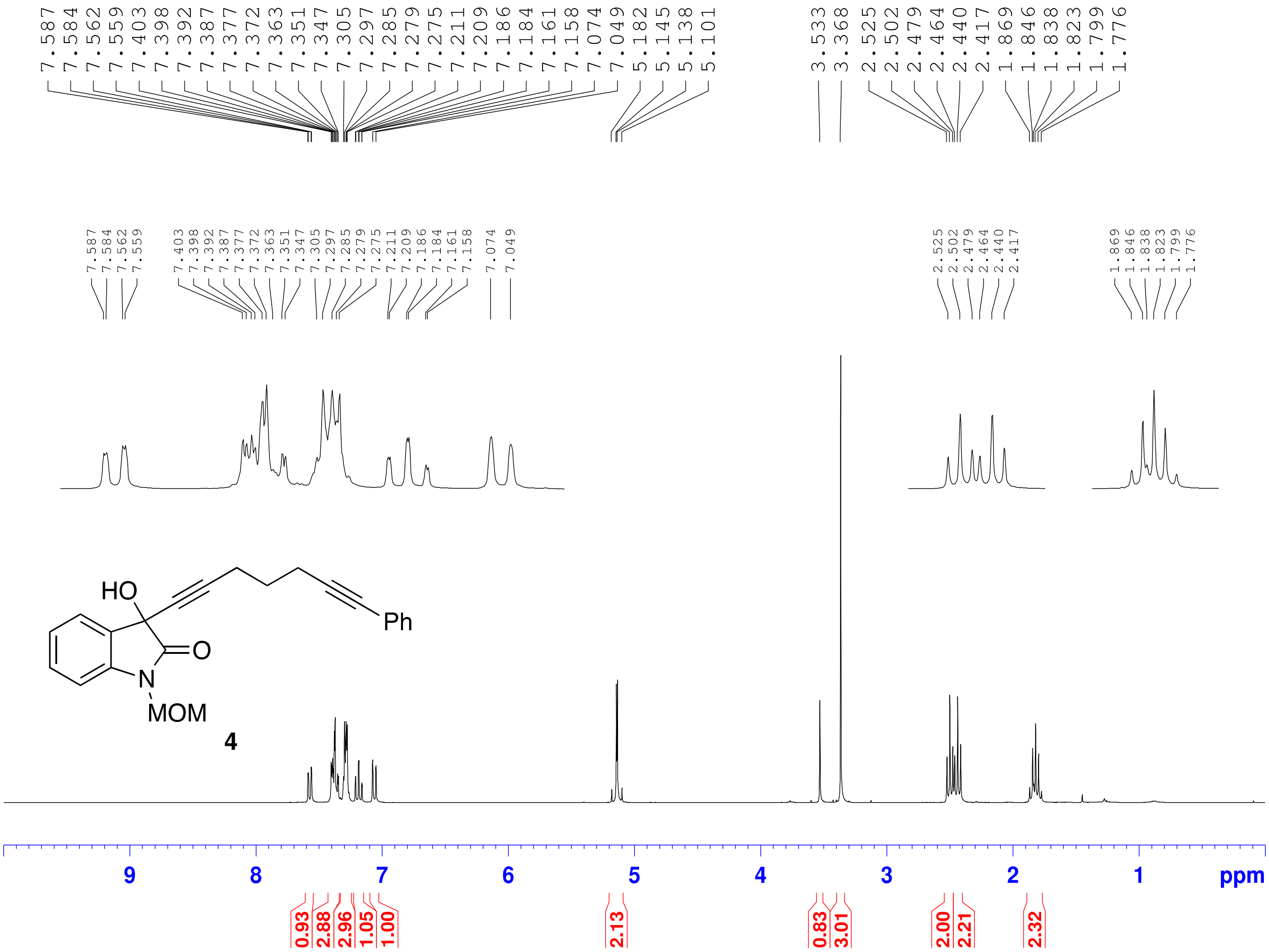

JM06-160 Crude
NMR 300

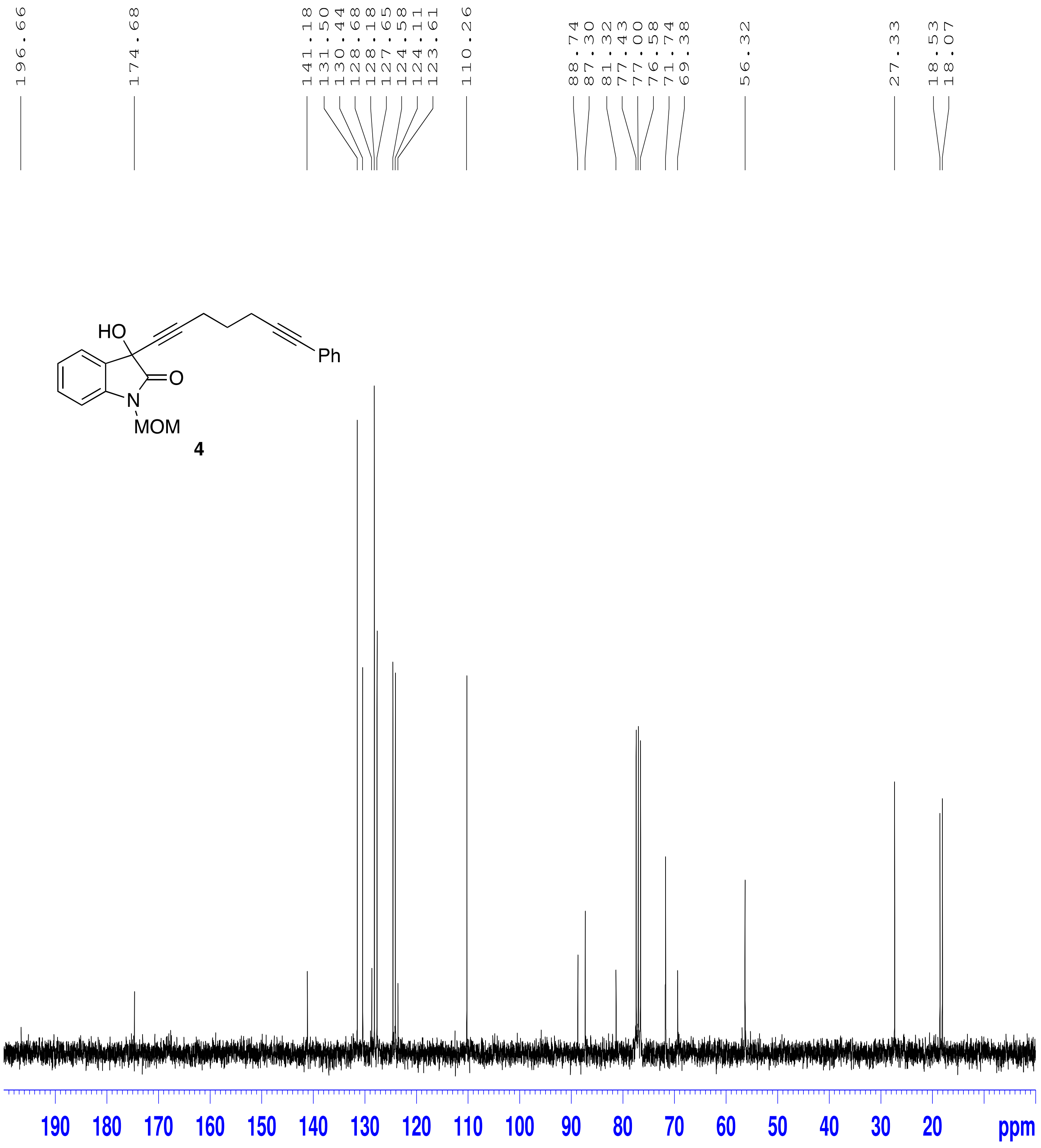

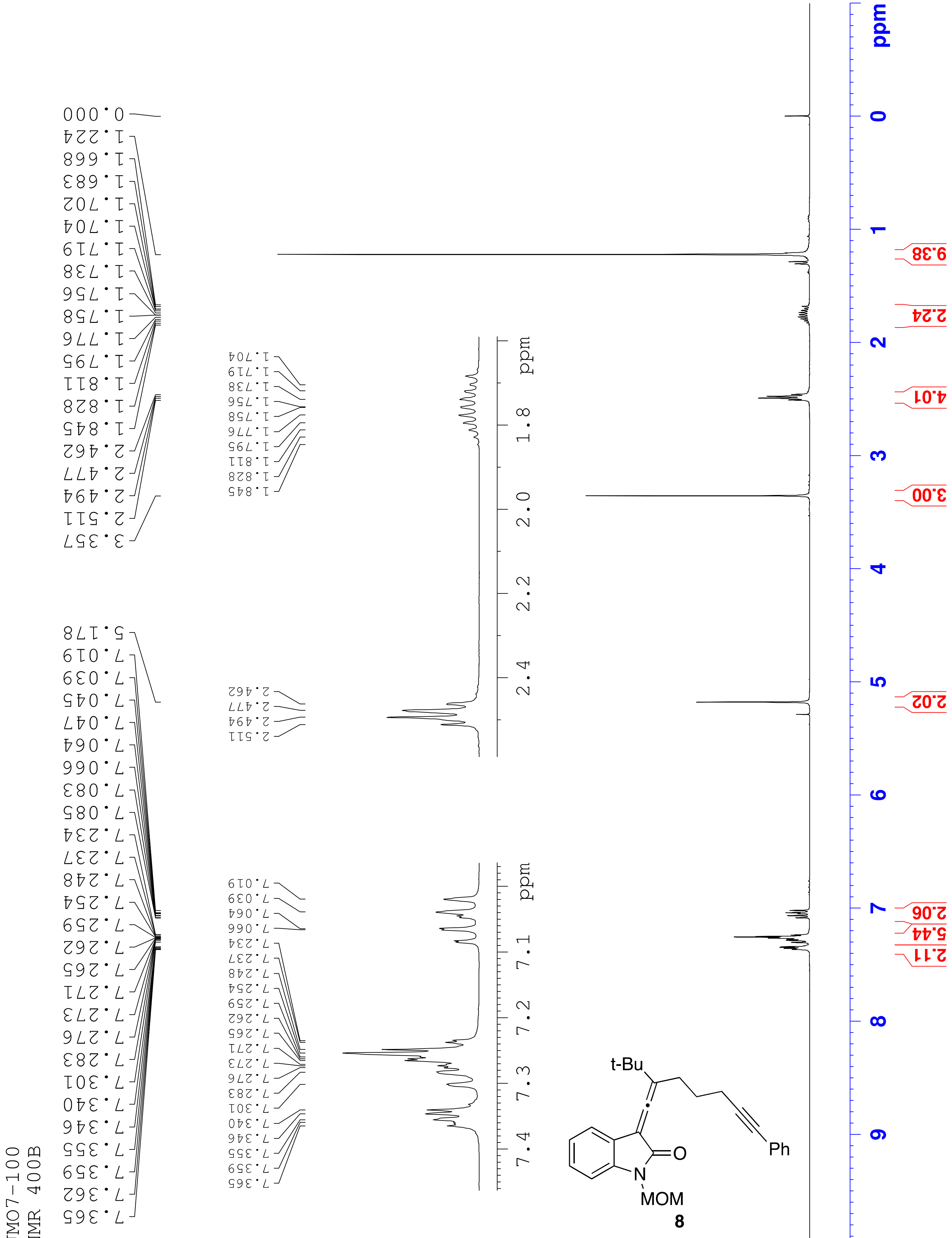
JMO7-100
NMR 400B
0.000
1.224
1.668
1.683
1.702
1.704
1.719
1.738
1.756
1.758
1.776
1.795
1.811
1.828
1.845
2.462
2.477
2.494
2.511
3.357
5.178
7.019
7.039
7.045
7.047
7.064
7.066
7.083
7.085
7.234
7.237
7.248
7.254
7.259
7.262
7.265
7.271
7.273
7.276
7.283
7.301
7.340
7.346
7.355
7.359
7.362
7.365
ppm
9.38
2.24
4.01
3.00
2.02
2.06
5.44
2.11
t-Bu
O
N
MOM
Ph
8

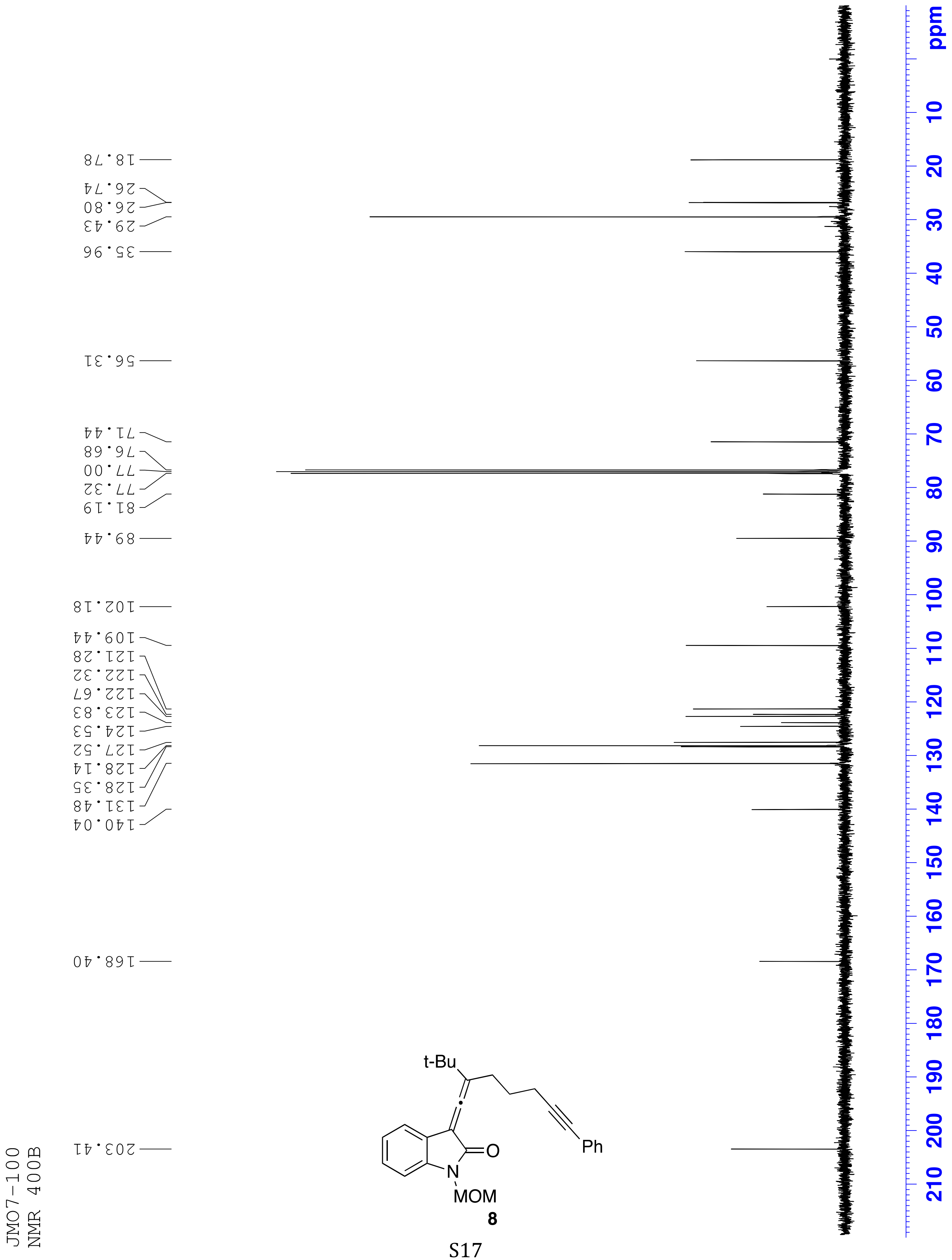
JMO7-100
NMR 400B
18.78
26.74
26.80
29.43
35.96
56.31
71.44
76.68
77.00
77.32
81.19
89.44
102.18
109.44
121.28
122.32
122.67
123.83
124.53
127.52
128.14
128.35
131.48
140.04
168.40
203.41
ppm
t-Bu
O
N
MOM
Ph
8

JMO7-116
NMR 400A
PROTON CDCl3 C:\Bruker\TOPSPIN brummond 2

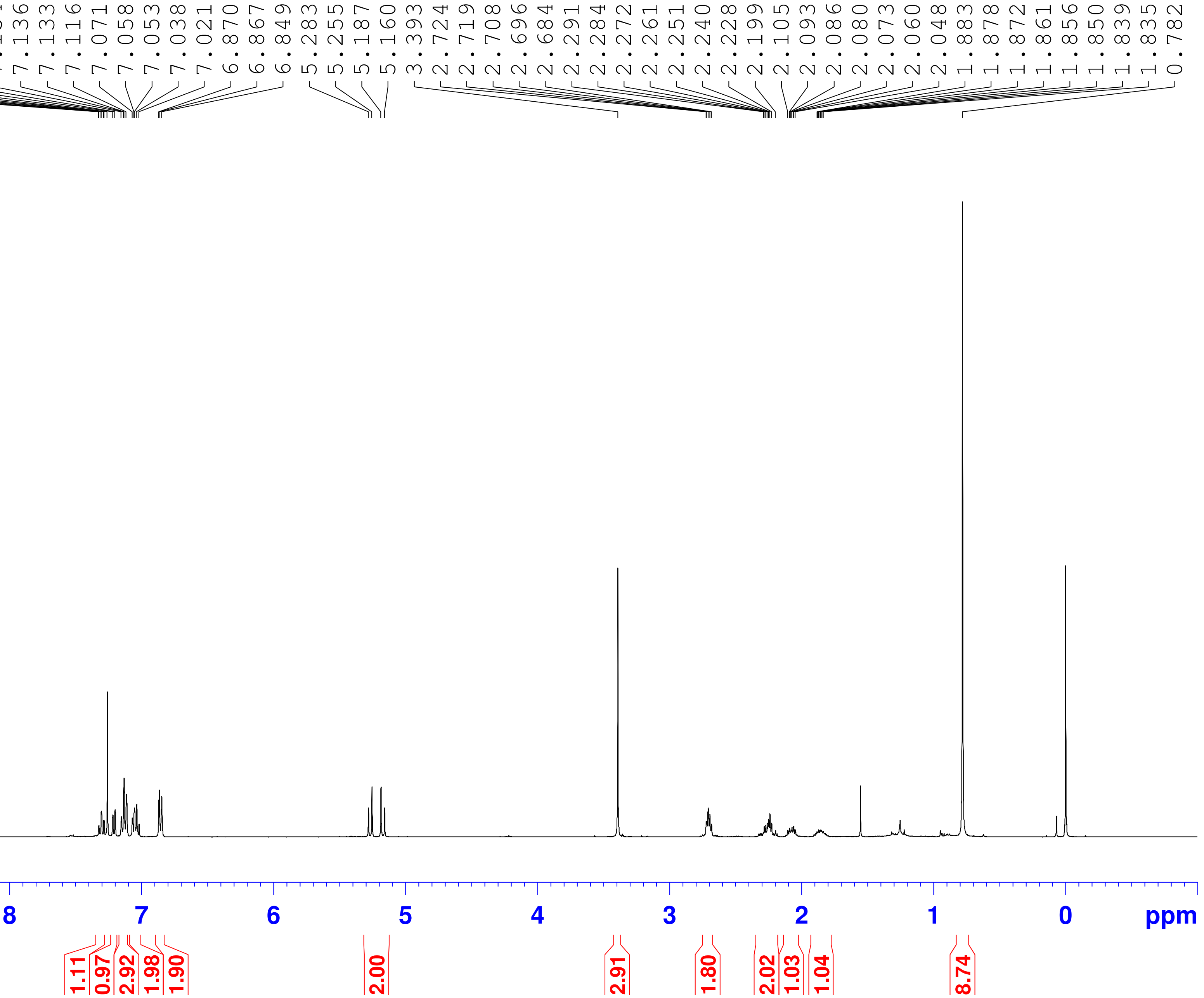

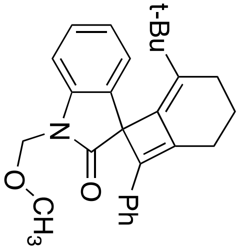

9

S18

JM07-124
NMR 300

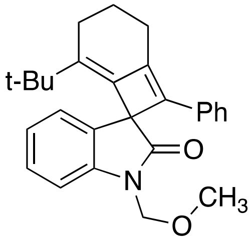

9

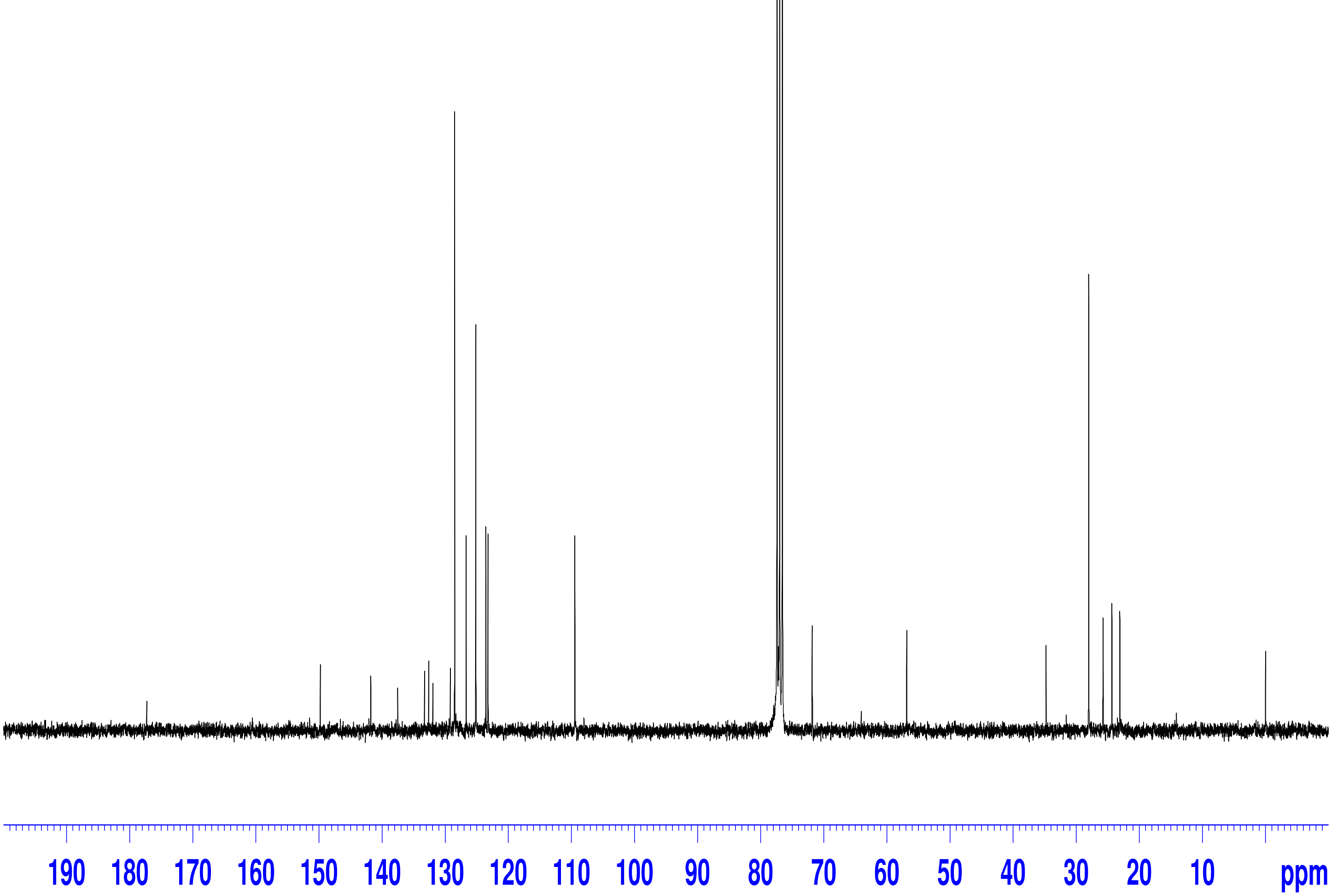

Supplement: File 1 — General methods, experimental and spectral data for all new compounds. [file Beilstein_J_Org_Chem-07-601-s001.pdf]
